# Diagnostic Value of Seven Different Imaging Modalities for Patients with Neuroblastic Tumors: A Network Meta-Analysis

**DOI:** 10.1155/2021/5333366

**Published:** 2021-09-01

**Authors:** Yu Wang, Yanfeng Xu, Ying Kan, Wei Wang, Jigang Yang

**Affiliations:** Department of Nuclear Medicine, Beijing Friendship Hospital, Capital Medical University, Beijing 100050, China

## Abstract

**Objective:**

We performed a systematic review and network meta-analysis (NMA) to compare the diagnostic value of seven different imaging modalities for the detection of neuroblastic tumors in diverse clinical settings.

**Methods:**

PubMed, Embase, Medline, and the Cochrane Library were searched to identify eligible studies from inception to Sep 29, 2020. Quality assessment of included studies was appraised with Quality Assessment of Diagnostic Accuracy Studies. Firstly, direct pairwise meta-analysis was conducted to calculate the pooled estimates of odds ratio (OR) and 95% confidence interval (CI) of the sensitivity, specificity, NPV, PPV, and DR. Next, NMA using Bayesian methods was performed. The superiority index was assessed to quantify the rank probability of a diagnostic test. The studies performed SPECT/CT or SPECT were analyzed separately from the ones only performed planar imaging.

**Results:**

A total of 1135 patients from 32 studies, including 7 different imaging modalities, were eligible for this NMA. In the pairwise meta-analysis, ^18^F-FDOPA PET/CT had a relatively high value of all the outcomes (sensitivity: 10.195 [5.332–19.493]; specificity: 17.906 [5.950–53.884]; NPV: 16.819 [7.033–40.218]; PPV: 11.154 [4.216–29.512]; and DR 5.616 [3.609–8.739]). In the NMA, ^18^F-FDOPA PET/CT exhibited relatively high sensitivity in all subgroups (all data: 0.94 [0.87–0.98]; primary tumor: 0.89 [0.53–1]; bone/bone marrow metastases: 0.96 [0.83–1]; and primary tumor and metastases (*P* + *M*): 0.92 [0.80–0.97]), the highest specificity in the subgroup of *P* + *M* (0.85 [0.61–0.97]), and achieved the highest superiority index in the subgroups of all data (8.57 [1–15]) and *P* + *M* (7.25 [1–13]).

**Conclusion:**

^18^F-FDOPA PET/CT exhibited the best diagnostic performance in the comprehensive detection of primary tumor and metastases for neuroblastic tumors, followed by ^68^Ga-somatostatin analogs, ^123^I-meta-iodobenzylguanidine (MIBG), ^18^F-FDG, and ^131^I-MIBG tomographic imaging.

## 1. Introduction

Neuroblastic tumors (NTs) are the most common extracranial solid tumors of children, which are derived from the primitive neural crest. NTs include neuroblastoma, ganglioneuroblastoma, and ganglioneuroma. Nearly 48% of neuroblastomas present with metastasis at the time of diagnosis [1, 2]. Therefore, accurate identification of all lesions is of importance for staging and establishing therapy protocol [3].

Imaging, especially nuclear medicine functional imaging, plays an indispensable role in the diagnosis, staging, surgical planning, response assessment, and follow-up of NTs. Since neuroblastic tumor cells specifically express the noradrenaline transporter, iodine radioisotope-labelled meta-iodobenzylguanidine (MIBG), a noradrenaline analogue, becomes an ideal tracer for imaging of the tumor lesions. MIBG was labelled with ^131^I at the beginning. Nowadays, ^123^I-labelled MIBG (^123^I-MIBG) is the mainstay of radiopharmaceutical in the diagnosis and management of NTs. Considering of the limitation in small lesions and prolonged acquisition time (24–48 hours) of the MIBG scan, positron emission tomography (PET) imaging is increasingly being applied in current clinical practice. In particular, when the tumor uptake of MIBG is weak or negative, ^18^F-fluorodeoxyglucose (FDG) PET imaging is recommended as a second-line imaging by the International Neuroblastoma Risk Group (INRG) guidelines [3] and the European Association of Nuclear Medicine (EANM) 2018 guidelines [2]. Various PET tracers have been utilized for imaging in neuroblastoma patients, including the metabolic compounds such as ^18^F-FDG and L-3,4-dihydroxy-6-^18^F-fluorophenylalanine (FDOPA), as well as the receptor-mediated compounds such as ^68^Ga-DOTA peptides and somatostatin analogues (SSAs) [4–6]. Traditional imaging, computed tomography (CT) and magnetic resonance imaging (MRI), also has an essential role in the staging and evaluation of surgical risks for the disease [7].

In the EANM 2018 guidelines for neuroblastoma, discussion is still ongoing on the effectiveness of various imaging modalities and the applicability of different tracers in diverse clinical settings. An increasing number of studies reported the utility of different imaging in the diagnosis of NTs. However, considering the radiation burden and imaging acquisition inconvenience to pediatric patients, head-to-head studies are few and most of them are of small sample sizes. Although there have been a few previous meta-analyses [8–10] of the diagnostic value of imaging modalities for NTs, there were some limitations in their data grouping. Moreover, all of them were conventional meta-analysis that evaluated one single imaging technique or simply compared two imaging modalities. Network meta-analysis (NMA) extends conventional meta-analysis, which is a novel synthesis of evidence. In contrast to the conventional pairwise meta-analysis, NMA draws together evidence from both direct and indirect comparison of multiple tests simultaneously [11]. NMA can calculate the effect size and quantify the rank probability of each diagnostic test between groups through indirect study comparison, even if there is no direct head-to-head study. Moreover, NMA with an arm-based model provides more natural variance-covariance matrix structures which make it more appropriate than the traditional meta-analysis. Therefore, we conducted an NMA and comprehensive systematic review to directly and indirectly compare the diagnostic value of all enrolled imaging modalities in NTs.

## 2. Materials and Methods

This systematic literature review and meta-analysis was performed according to the “Preferred Reporting Items for Systematic Reviews and Meta-Analyses” (PRISMA) guidelines and was registered in PROSPERO (CRD42020206862).

### 2.1. Search Strategy

A systematic literature search was conducted based on the Population, Interventions, Comparator, Outcome, and Study design (PICOS) principle. PubMed, the Cochrane Library, Embase, and Medline were searched from inception to Sep 29, 2020.

### 2.2. Inclusion and Exclusion Criteria

Inclusion criteria were as follows: (1) population: patients diagnosed with NTs, (2) intervention: any type of imaging modality was performed, (3) comparator: compared to each other, (4) outcomes: sensitivity, specificity, positive predictive value (PPV), and negative predictive value (NPV), and (5) study type: diagnostic accuracy study.

Exclusion criteria were as follows: (a) case reports, reviews, meeting abstracts, or comments; (b) non-human studies or non-English articles; (c) sample size ＜ 10; (d) lack of essential data, including true positive (TP), true negative (TN), false positive (FP), and false negative (FN) values; (e) study enrolling recurrent or refractory patients, and (f) overlapping patients reported. Once articles enrolling overlapping patients were identified, the recently published article with more patients was included.

### 2.3. Data Extraction

Two researchers independently performed the literature searching, screening, and data extracting. The differences were discussed until reaching consensus. The following information was collected: basic information of studies (first author, publication year, study period, original country, study design, and follow-up time), patient characteristics (sample size, age, and gender), type of lesions (P, primary tumor; BM, bone and bone marrow metastases; and *P* + *M*, primary tumor and metastases), type of imaging modality, standard reference, and raw diagnostic data (TP, FP, TN, and FN).

### 2.4. Quality Assessment

The quality of enrolled studies was appraised with Quality Assessment of Diagnostic Accuracy Studies (QUADAS-2) [12] by two authors. Discrepancies between the authors were resolved by discussion. The QUADAS-2 includes four domains: patient selection, reference standard, index text, and flow and timing. Each domain is assessed in terms of risk of bias, and the first 3 domains are also appraised in terms of concerns regarding applicability. RevMan (Version 5.3.5, the Cochrane Collaboration, Oxford, UK) was used to conduct the assessment.

### 2.5. Statistical Analysis and Data Synthesis

Traditional pairwise meta-analysis was conducted to calculate the pooled estimates of odds ratio (OR) and 95% confidence interval (CI) of sensitivity, specificity, NPV, PPV, and detection rate (DR) of various imaging modalities. Heterogeneity was assessed by the *χ*^2^ test and *I*^2^ statistics. A fixed-effect model would be applied if *P* > 0.1 and/or I^2^< 50%. Otherwise, a random-effect model would be conducted. Subgroup analyses were conducted based on diverse clinical settings. The publication bias was assessed by Deeks' funnel plot asymmetry test. Traditional meta-analyses were performed using STATA (version 15.0, StataCorp, College Station, TX).

Next, the evidence network structure was performed with package gemtc (v 0.8–8) in *R* software (Version 4.0.3, Comprehensive *R* Archive Network). Each node stands for a different diagnostic test, and the thickness of lines between nodes represents the number of studies that directly compared the two tests. Then, Bayesian NMA was performed by an arm-based model, which was developed by Nyaga et al. [13]. We run three chains in parallel until there was convergence. Trace plots were applied to visually check whether the distributions of the three simulated chains were mixed properly and were stationary. The superiority index [13] was estimated to quantify the rank probability of a diagnostic test. The larger the superiority index was, the more accurately a test was expected to predict the targeted condition compared to other screening tests. A two-sided *P* value  < 0.05 was considered statistically significant in all statistical tests. All NMA were performed using *R* software, package rstan (v 2.21.2), package loo (v 2.3.1), and package plyr (v 1.8.6).

## 3. Results

### 3.1. Literature Search Results

**Figure 1:**
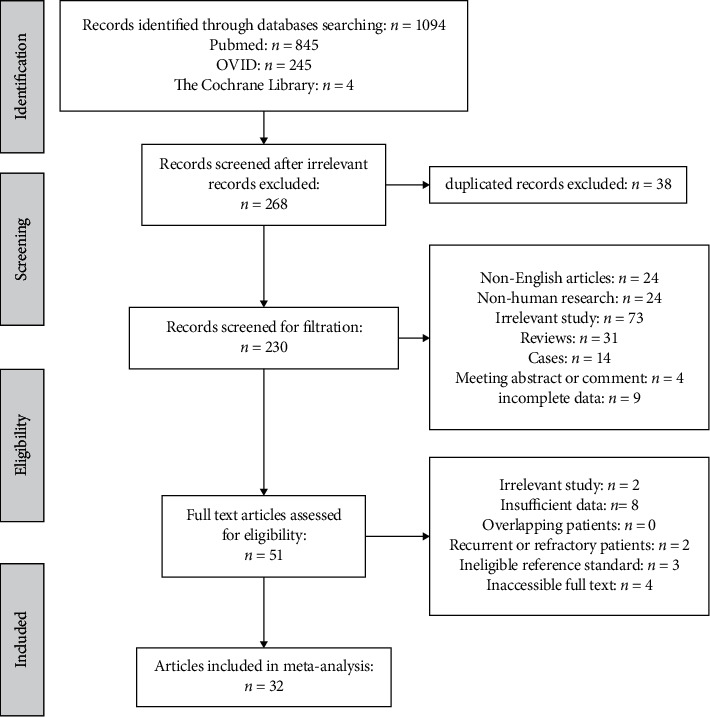
Flowchart for the selection of studies for meta-analysis.

A total of 1,094 studies were initially retrieved. After excluding irrelevant articles (*n* = 826) and duplicated records (*n* = 38), the remaining 230 studies were further assessed. A total of 51 studies were evaluated for eligibility by full-text review, after excluding non-English articles (*n* = 24), non-human studies (*n* = 24), irrelevant studies (*n* = 73), reviews (*n* = 31), cases (*n* = 14), meeting abstracts or comments (*n* = 4), and the studies with incomplete data (*n* = 9). After full-text review, irrelevant studies (*n* = 2), studies with insufficient data (*n* = 8) or ineligible reference standard (*n* = 3), studies focusing on recurrent or refractory patients (*n* = 2), and inaccessible full text (*n* = 4) were ruled out. Finally, thirty-two diagnostic studies [14–45] met the inclusion criteria (Figure 1).

### 3.2. Characteristics of Included Studies and Quality Assessment

**Figure 2:**
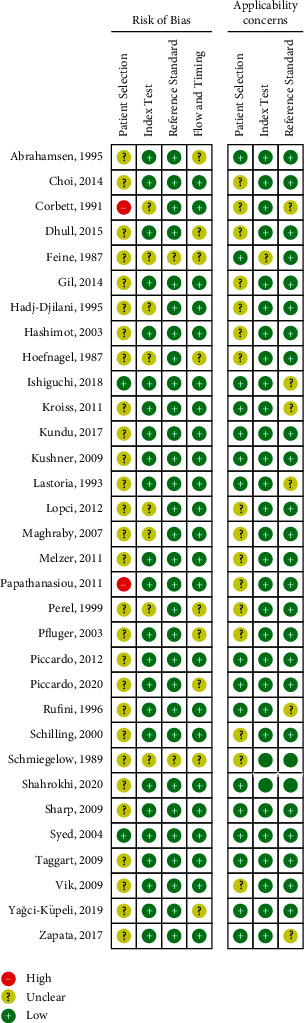
QUADAS-2 methodological quality summary.

A total of 1135 patients from 32 studies, including 7 different imaging modalities, were eligible for this NMA. Nine (28.1%) [14, 15, 23, 25, 28, 30, 31, 35, 39] of 32 studies were prospective design. Nineteen studies [14, 15, 17, 18, 20-24, 26, 29, 31, 36, 39–42, 44, 45] included at least two tests (imaging methods), and 14 (73.7%) of them were head-to-head studies. Twenty (62.5%) [15, 18, 20–26, 29-31, 33, 34, 36, 40-43, 45] of 32 studies investigated ^123^I-MIBG imaging, nine (28.1%) studies [14, 17, 26, 27, 36, 37, 39, 42, 44] focused on ^131^I-MIBG, eleven (34.4%) studies [16-20, 23, 24, 38–40, 42] performed ^18^F-FDG-PET or PET/CT, three (9.4%) studies [15, 21, 22] addressed ^18^F-FDOPA, six (18.8%) studies [18, 21, 24, 29, 44, 45] evaluated CT or MRI, two (6.3%) studies [14, 41] assessed ^68^Ga- SSAs, and one (3.1%) study [31] inquired into ^111^In-pentetreotide. The remaining four (12.5%) studies [33, 35, 36, 45] mixed ^131^I-MIBG and ^123^I-MIBG together, and all of them acquired planar imaging only. All ^18^F-FDOPA studies and one of the ^68^Ga-SSAs [14] acquired imaging with an integrated PET/CT scanner. Among the FDG studies, ten of them conducted with PET/CT, while the remaining one [24] performed with a dedicated PET scanner. Among ^123^I-MIBG studies, 7 studies only performed planar imaging, 8 studies acquired single-photon-emission computed tomography (SPECT) imaging, and 5 studies applied SPECT/CT. In the nine ^131^I-MIBG studies, only 2 studies performed with the SPECT/CT scanner, whereas the other 7 studies acquired planar imaging. In the six CT or MRI studies, 3 studies conducted with MRI, 2 studies combined the results of CT and MRI together, and the last one performed CT. The study focused on ^111^In-pentetreotide-only-acquired planar imaging. The principal characteristics of all included studies were displayed in Table 1. QUADAS-2 score results from each study are presented in Figure 2.

### 3.3. Outcome of Pairwise Meta-Analysis

A direct paired comparison of the 7 different imaging modalities of the diagnostic value for NTs was performed. The estimated OR and 95% CI of the sensitivity, specificity, NPV, PPV, and DR are summarized in Table 2. In both sensitivity and specificity, ^18^F-FDOPA had a relatively good performance of all outcomes (sensitivity: 10.195 [5.332–19.493]; specificity: 17.906 [5.950–53.884]; NPV: 16.819 [7.033–40.218]; PPV: 11.154 [4.216–29.512]; and DR 5.616 [3.609–8.739]).

When stratified according to clinical settings, ^123^I-MIBG imaging showed the highest DR for the diagnosis of primary tumor (9.486 [3.484–25.826]). For the detection of bone and bone marrow metastases, CT or MRI exhibited the highest DR (7.170 [4.503–11.415]), followed by ^18^F-FDOPA (5.283 [2.325–12.005]). Regarding the detection of primary tumor and metastases (*P* + *M*), ^131^I-MIBG exhibited the highest DR (22.563 [8.020–63.480]) and ^18^F-FDOPA had the second highest DR (5.943 [3.480–10.149]) (Table 3).

### 3.4. Evidence Network

**Figure 3:**
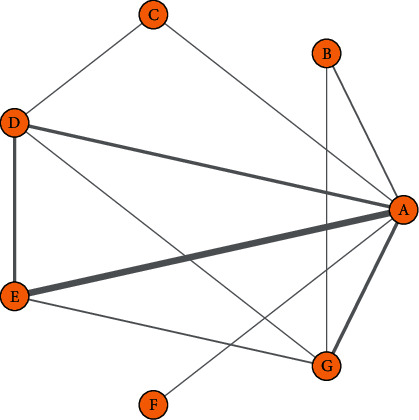
Evidence network of the 7 imaging modalities.

The evidence network included seven imaging modalities. The result revealed the number of studies investigating ^123^I-MIBG was the highest. Studies comparing ^123^I-MIBG with ^18^F-FDG were the most, followed by ^131^I-MIBG, CT or MRI and ^18^F-FDOPA imaging (Figure 3).

### 3.5. Outcomes of Network Meta-Analysis and Ranking of Diagnostic Tests

**Figure 4:**
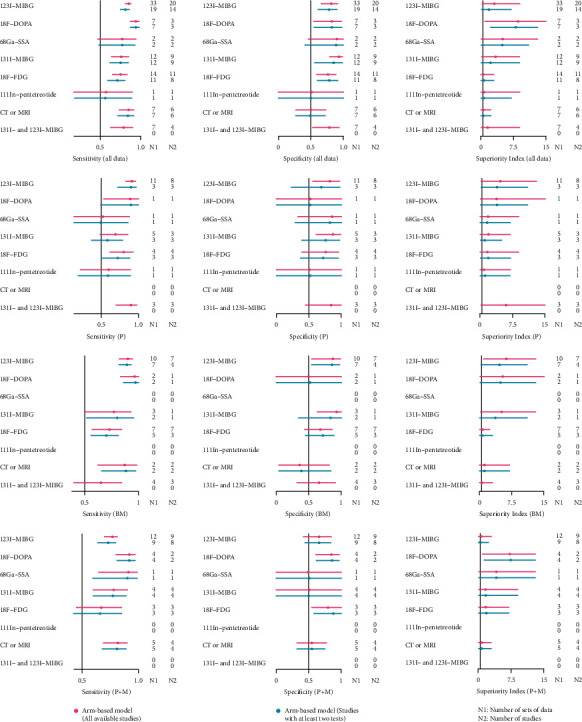
Network meta-analysis of all data and subgroups.

The results of the NMA for the seven imaging methods are shown in Figure 4. ^18^F-FDOPA exhibited relatively high sensitivity in all subgroups (all data: 0.94 [0.87–0.98]; primary tumor: 0.89 [0.53–1]; BM: 0.96 [0.83–1]; and *P* + *M*: 0.92 [0.80–0.97]) and the highest specificity in the subgroup of *P* + *M* (0.85 [0.61–0.97]). ^131^I-MIBG exhibited the highest specificity in the subgroups of all data (0.93 [0.79–0.99]), primary tumor (0.87 [0.61–0.99]), and BM (0.93 [0.63–1]). According to the superiority index, ^18^F-FDOPA achieved the highest value in the subgroups of all data (8.57 [1–15]) and *P* + *M* (7.25 [1–15]). ^131^I- and ^123^I-MIBG subgroup had the highest superiority index in the diagnosis of primary tumor (6.06 [0.33–15]). ^123^I-MIBG ranked the highest superiority index in the subgroup of BM (6.27 [1–15]).

**Figure 5:**
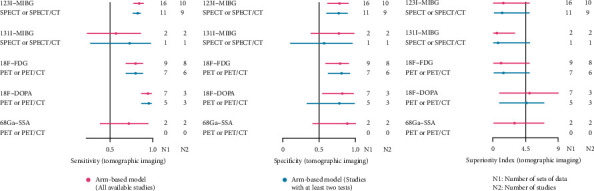
Network meta-analysis of tomographic imaging.

After removing the studies only conducted with planar imaging and traditional imaging (Figure 5), ^18^F-FDOPA PET/CT achieved the highest sensitivity (0.94 [0.87–0.98]) and superiority index (5.06 [1–9]), and ^68^Ga-SSAs PET or PET/CT reached the highest specificity (0.89 [0.42–1]).

### 3.6. Publication Bias

The results of assessment of publication bias demonstrated symmetrical distribution in all subgroups except the tomographic imaging subgroup (*P*=0.02). Deeks' funnel plots were presented in the supplementary materials (Figures S1–5)

## 4. Discussion

The current NMA reveals that ^18^F-FDOPA PET/CT should be the imaging modality of choice for the comprehensive detection of primary tumor and metastases in NTs. ^123^I-MIBG SPECT or SPECT/CT present satisfactory performance for the diagnosis of both primary tumor and BM.

The EANM 2018 guidelines focus on SPECT (^123^MIBG) and PET (PET/CT with ^18^F-FDG, ^18^F-FDOPA, and ^68^Ga-DOTA peptides) tracers currently used in clinical practice. These guidelines presented general indications, advantages, and limitations along with recommendations on imaging protocols, interpretation of findings, and reporting results for nuclear medicine imaging in neuroblastoma. However, discussion regarding the clinical settings that may benefit most from the use of one tracer over the others is still ongoing. It is well known that the accumulation and decarboxylation of L-DOPA in neuroendocrine tumors (NETs) make it an excellent tracer for catecholamine metabolism in NETs, including NTs. ^18^F-FDOPA has already been applied in the diagnosis of pheochromocytoma (PCCs) [46, 47] and recommended as a first-line PET/CT tracer for the detection of medullary thyroid carcinoma [48]. The EANM 2018 guidelines also suggested ^18^F-FDOPA may currently be the best PET tracer alternative to ^123^I-MIBG for the assessment of NTs [2]. It showed a remarkable performance in the diagnosis of neuroblastoma in the current meta-analysis as well as other existing research [15, 22, 49]. In the present NMA, ^18^F-FDOPA PET/CT exhibited relatively higher sensitivity in all clinical settings, the highest sensitivity, and specificity in the subgroup of *P* + *M*, which ranked the first according to superiority index. Therefore, ^18^F-FDOPA PET/CT may become a promising diagnostic tool for neuroblastoma in the future. ^68^Ga-SSAs can bind to specific somatostatin receptors on the cell surface of NETs, which is also an imaging tracer of choice in NETs [50]. ^68^Ga-SSAs PET or PET/CT ranked the second in the diagnostic value of NTs. Nevertheless, there are only 2 studies focused on this radiopharmaceutical. The study from Pezhman [14] suggested ^68^Ga-SSAs PET/CT was superior to ^131^I-MIBG SPECT/CT in providing valuable information for both primary staging and follow-up in patients with neural crest tumors, including NTs and PCCs. Another study [41] did not enroll TN patients, and the specificity cannot be evaluated. So, the results should be interpreted cautiously. Interestingly, both ^18^F-FDOPA and ^68^Ga-SSAs are relatively new tracers for NETs, and a number of studies which did comparison between them in the detection of neuroendocrine tumors and other diseases have been reported [51, 52]. However, the study that head to head compares ^18^F-FDOPA and ^68^Ga-SSAs in patients with neuroblastic tumors has not been found. Thus, further study is expected.

^123^I-MIBG imaging, the most commonly utilized molecular imaging modality for the identification and prognostic evaluation of neuroblastoma, exhibited a relatively high value in both sensitivity and specificity for the detection of primary tumor and metastases. In particular, ^123^I-MIBG achieved the highest superiority index in the detection of BM. After excluding the studies only performed with planar imaging, ^123^I-MIBG SPECT or SPECT/CT exhibited a higher superiority index compared with ^18^F-FDG-PET or PET/CT and ^131^I-MIBG SPECT or SPECT/CT, which is consistent with the EANM guidelines of 2018 [2]. Thus, we propose that it is highly necessary to perform ^123^I-MIBG imaging with SPECT/CT.

The current NMA showed that ^18^F-FDG was inferior to ^18^F-FDOPA, ^123^I-MIBG, or ^131^I-MIBG in the separate evaluation of both primary tumor and BM. ^18^F-FDG, a glucose analogue, concentrates at lesions with increased glucose metabolism, including most tumors and inflammation or infection. Therefore ^18^F-FDG is less specific for NTs than MIBG or FDOPA. Notably, ^18^F-FDG displayed a higher superiority index compared with MIBG when it was employed to comprehensively evaluate the primary tumor and metastases in the whole body. That may be related with the higher resolution of PET than SPECT. The study of Henriette [24] reported the false negative results of ^123^I-MIBG were due to small lesion size (mean lesion diameter 1.7 cm) and low uptake. Combined with our results, it suggested that ^18^F-FDG could play a complementary role of MIBG when the lesion is small or non-MIBG avid. Additionally, beyond lesion recognition,^18^F-FDG may be helpful in tumor staging, treatment evaluation, and prognostic assessment of neuroblastoma [40, 42, 53]. Higher FDG uptake was observed in patients with higher-stage MYCN amplification [54] or advanced stage [55]. Maximum standardized uptake value (SUVmax) was all significantly higher in patients with worse overall survival [54].

^131^I-MIBG imaging exhibited moderate diagnostic characteristics based on the superiority index. It may be limited by the unfavorable imaging characteristics of the isotope ^131^I. Nowadays, considering higher radiation dose of ^131^I-MIBG compared with ^123^I-MIBG, many studies [4, 56] recommend that diagnostic ^131^I-MIBG was indicated only when ^123^I-MIBG is unavailable or ^131^I-MIBG therapy is contemplated. Preliminary studies of ^124^I-MIBG [57, 58] as well as ^18^F-meta-fluorobenzylguanidine (^18^F-MFBG) [59, 60] and MIBG variants [61] (^18^F-fluoropropylbenzylguanidine, ^18^F-FPBG) are ongoing. Those imaging agents are proved to improve image quality and demonstrate promising performance in the diagnosis of NTs.

CT and MR are widely available and routinely used in clinical practices, whereas they only showed moderate sensitivity and low specificity in the detection of BM in the NMA. The high incidence of false positive findings was probably related with the fact that the traditional imaging modality cannot distinguish posttherapy bone marrow changes from residual tumor. In recent years, increasing studies suggested that whole-body “diffusion-weighted imaging with background body signal suppression” is feasible for assessment of the primary lesions [62, 63] and lymph node metastases [18] of NTs. However, according to our results, it should be carefully used in NT patients due to its low specificity in the identification of skeleton lesions. Regarding the diagnostic value of ^111^In-pentetreotide, only one study [31] was included, and the data of this study are incomplete. Therefore, more high-quality studies are expected.

Three previous meta-analyses of the diagnostic value of different imaging modalities for NTs were identified. All of them evaluated one single imaging technique [8, 9] or simply compared two imaging modalities [10]. Moreover, the studies conducted with SPECT/CT and planar imaging were not analyzed separately in the meta-analysis from the work of Jia X et al. [10], which was irrational. Because there is a huge difference between planar imaging and SPECT/CT in imaging resolution, there is little comparability between them. Li et al. [9] attempted to assess the diagnostic accuracy of PET(CT) in patients with neuroblastoma in their meta-analysis, but they did not calculate the data of FDG-PET(CT) and FDOPA PET(CT) separately. Therefore, the reference value of this study for clinical practice is fairly limited.

This NMA has several limitations. Firstly, subgroup analyses were not conducted based on lesion-based analysis versus patient-/scan-based analysis because in the subgroup of primary tumor, all included studies were patient-/scan-based analysis. In the other two subgroups, most of the enrolled studies were lesion-based analysis, and patient-/scan-based analyses were performed in only 9 studies for the evaluation of 6 imaging modalities. Secondly, due to the small number of included studies for each imaging modality, subgroup analyses were not performed according to other variables such as study design, patients' baseline characteristics, interval between injection and acquisition, and other imaging protocols. Thirdly, CT and MR were not analyzed separately. In the included studies, half (3/6) of the included studies mixed CT and MR together to compare with nuclear medicine imaging. Only two studies investigated the performance of MR, and just one study reported the data of CT. Finally, the estimated specificity of several groups displayed a wide range of 95% credible intervals (0–1), such as ^18^F-FDOPA in the BM and P subgroups, ^111^In-pentetreotide in the P subgroup, and ^68^Ga-SSAs and ^131^I-MIBG in the *P* + *M* subgroup. This demonstrates that the specificity was unavailable because some studies did not enroll TN cases. Further direct comparative studies with standardized data would be necessary.

## 5. Conclusions

In conclusion, ^18^F-FDOPA PET or PET/CT exhibited the best diagnostic performance in the comprehensive detection of primary tumor and metastases in NTs, followed by ^68^Ga-SSAs, ^123^I-MIBG, ^18^F-FDG, and ^131^I-MIBG tomographic imaging. ^123^I-MIBG SPECT or SPECT/CT present satisfactory performance for the diagnosis of both primary tumor and BM. Further comparative studies with standardized data are expected in the future.

## Figures and Tables

**Table 1:** Characteristics of the enrolled studies.

Study	Study period	Country	Study design	Head to head	No. of patients (M : F)	Age (years)	Follow-up time (months)	Lesion	Nuclear imaging	Reference standard

Piccardo et al. [15]	2013.12–2017.1	Italy	Pro	Yes	18 (12 : 6)	2.8 ± 1.6 (1–6)	29.3 (19–53)	P; BM	^123^I-MIBG SPECT/CT; ^18^F-FDOPA PET/CT	BMB pathology and CT and/or MRI follow-up
Shahrokhi et al. [14]	NR	Iran	Pro	Yes	15 (4 : 11)	2–52	NR	P	^123^I-MIBG SPECT/CT; ^68^Ga-DOTATATE PET/CT	BMB pathology and FDG-PET/CT follow-up
Yağcı-Küpeli et al. [16]	2014.7–2017.12	Turkey	Retro	NR	15 (8 : 7)	4 (1–14)	≥6	BM	^18^F-FDG-PET/CT	BMB pathology and FDG-PET/CT follow-up
Ishiguchi et al. [18]	2012.6–2016.1	Japan	Retro	Yes	13 (7 : 6)	2.9 ± 2.0	NR	BM	^18^F-FDG-PET/CT; ^123^I-MIBG SPECT; whole-body DWIBS	Histopathology, ^123^I-MIBG SPECT/CT, PET/CT, and BS
Zapata et al. [38]	2009.1–2014.10	America	Retro	NR	20 (8 : 12)	3.8 (0.5–18)	NR	BM	^18^F-FDG-PET/CT	BMB pathology and ^18^F-FDG-PET/CT
Kundu et al. [39]	NR	India	Pro	Yes	18	NR	NR	P	^18^F-FDG-PET; ^131^I-MIBG planar	Histopathology; CT
Dhull et al. [17]	2007.6–2012.12	India	Retro	NR	28	5.5 ± 5.6	≥6	P	^18^F-FDG-PET/CT; ^131^I-MIBG SPECT	Histopathology and/or clinical/imaging follow-up
Choi et al. [19]	2003.1–2010.8	Korea	Retro	NR	30 (18 : 12)	2.7	NR	P	^18^F-FDG-PET/CT	Histopathology; clinical and imaging (^123^I-MIBG scans,^99m^Tc-MDP BS) follow-up
Gil et al. [20]	2005.11–2013.1	Korea	Retro	Yes	8 (3 : 5)	3.5 (2–5)	≥6	P; BM	^18^F-FDG-PET/CT; ^123^I-MIBG SPECT	Histopathology; clinical and imaging (^123^I-MIBG scans, ^99m^Tc-MDP BS) follow-up
Piccardo et al. [22]	NR	Italy	Retro	Yes	19 (4 : 15)	1–41	≥4	*P* + *M*	^123^I-MIBG SPECT; ^18^F-FDOPA PET/CT	CT, MRI, histopathology, and clinical follow-up
Lopci et al. [21]	NR	Italy	Retro	Yes	21 (4 : 7)	7.4 (0.3–38)	NR	*P* + *M*	^18^F-FDOPA PET/CT; CT or MRI	Histopathology; clinical and imaging (^123^I-MIBG) follow-up
Papathanasiou et al. [22]	2004.11–2008.10	United Kingdom	Pro	Yes	28 (16 : 12)	7.5 (2–45)	NR	BM	^18^F-FDG-PET/CT; ^123^I-MIBG with/without SPECT/CT	Pathology and clinical follow-up
Melzer et al. [24]	2004.7–2010.7	Germany	Retro	Yes	19 (10 : 9)	5.9 (0.7–19.1)	≥6	*P* + *M*	^18^F-FDG-PET; ^123^I-MIBG SPECT; CT or MRI	Histopathology; clinical and imaging (^123^I-MIBG/FDG-PET/MR/CT) follow-up
Kroiss et al. [41]	NR	Austria	Retro	Yes	5 (0 : 5)	3–62	NR	*P* + *M*	^68^Ga-DOTA-TOC; ^123^I-MIBG SPECT	Histopathology; CT and MRI
Sharp et al. [40]	2003.1–2007.10	America	Retro	Yes	60 (37 : 23)	3.1	NR	*P* + *M*	^123^I-MIBG planar + SPECT; ^18^F-FDG-PET/CT	Urine catecholamines, histopathology, MIBG, and FDG-PET
Vik et al. [25]	NR	America	Pro	NR	100 (57 : 43)	4.7 ± 6.9 (0.08–58)	NR	P	^123^I-MIBG planar with/without SPECT/CT	Pathology and clinical follow-up
Taggart et al. [42]	2001–2005	America	Retro	Yes	14	1–30	NR	BM	^123^I-MIBG planar; ^131^I-MIBG planar; ^18^F-FDG-PET	Urine catecholamines, histopathology, MIBG, and ^18^F-FDG-PET
Kushner et al. [26]	NR	America	Retro	No	113 (70 : 43)	NR	18 (5–53)	*P* + *M*	^131^I-MIBG planar; ^123^I-MIBG planar	Histopathology, CT, bone scan, bone marrow biopsy, and follow-up
Maghraby et al. [28]	NR	Saudi Arabia	Pro	NR	21 (13 : 8)	4.13 ± 4.06 (0.17–6)	NR	P	^123^I-MIBG planar	Histopathology; clinical follow-up
Syed et al. [27]	1996–1999	Pakistan	Retro	NR	26 (18 : 8)	5 ± 3 (0.7–17)	12–36	P; BM	^131^I-MIBG planar	Histopathology; clinical and imaging (^123^I-MIBG scans,^99m^Tc-MDP BS; CT; and US) follow-up
Hashimot et al. [30]	NR	Japan	Pro	NR	33 (20 : 13)	0.6 (0.5–0.83)	NR	P	^123^I-MIBG planar	Histopathology; clinical and imaging (^123^I- or^131^I-MIBG) follow-up
Pfluger et al. [29]	NR	Germany	Retro	Yes	28 (18 : 10)	3.2 (0.1–11)	≥6	*P* + *M*	^123^I-MIBG SPECT; MRI	Histopathological findings or follow-up control examinations
Schilling et al. [31]	NR	Germany	Pro	NR	88 (48 : 40)	1.2 (0.1–24.2)	38 (median)	P	^123^I-MIBG planar;^111^In-pentetreotide planar	BMB pathology and CT and/or MRI follow-up
Perel et al. [32]	1985–1996	France	Retro	NR	30	NR	38 (7–120)	P	^131^I- and ^123^I-MIBG planar	BMB pathology and FDG-PET/CT follow-up
Rufini et al. [43]	1991.5–1994.12	Italy	Retro	Yes	29 (19 : 10)	2.6 (0.17–12)	NR	P; BM; *P* + *M*	^123^I-MIBG planar and SPECT	Histopathology, CT, MR, US, bone scan, and bone marrow biopsy
Hadj-Djilani et al. [34]	NR	Switzerland	Retro	NR	27 (19 : 8)	3.5 (0.03–24)	NR	P	^123^I-MIBG planar	Histopathology
Abrahamsen et al. [33]	1984.9–1985.12	Denmark	Retro	NR	36 (20 : 16)	3 (0.1–14.8)	NR	P	^131^I- and ^123^I-MIBG planar	BMB pathology and ^18^F-FDG-PET/CT
Lastoria et al. [44]	NR	Italy	Retro	NR	28 (18 : 10)	2.4 ± 2.1	NR	*P* + *M*	^131^I-MIBG planar; CT	Histopathology; clinical and ^99m^Tc-MDP imaging (bone involvement)
Corbett et al [45]	NR	United Kingdom	Retro	No	19 (9 : 10)	4.8 (0.5–13.6)	NR	BM	^123^I-MIBG planar; MRI	Histopathology (bone marrow aspirate) and imaging (^123^I-MIBG, MRI, and ^99m^Tc-MDP)
Schmiegelow et al. [35]	NR	Finland	Pro	NR	96 (57 : 39)	2 (0.9–5.6)	NR	P; BM	^131^I- and ^123^I-MIBG planar	Histopathology; clinical and imaging (^123^I-MIBG scans, and ^99m^Tc-MDP BS) follow-up
Hoefnagel et al. [37]	NR	The Netherlands	Retro	NR	94 (50 : 44)	0–52	NR	P	^18^F-FDG-PET/CT; ^123^I-MIBG planar with/without SPECT/CT	Pathology and clinical follow-up
Feine et al. [36]	1985–1987	Germany	Retro	No	36	NR	NR	P; BM; *P* + *M*	^131^I-MIBG planar	Histopathology, clinical follow-up

Retro, retrospective; Pro, prospective; NR, not reported; P, primary tumor; BM, bone and bone marrow metastases; *P* + *M*, primary tumor and metastases; DWIBS, diffusion-weighted imaging with background body signal suppression; BS, bone scan; BMB, bone marrow biopsy; CECT, contrast-enhanced CT; US, ultrasound.

**Table 2:** Pairwise meta-analysis for sensitivity, specificity, NPV, PPV, and DR of NTs.

Included studies	Comparisons	Heterogeneity assessment		Pairwise meta-analysis		
		*I* ^2^	*P*_*h*_	OR (95%CI)	*Z*	*P*

*Sensitivity*						
2 studies	^18^F-FDOPA vs. ^123^I-MIBG	0.0%	0.887	7.458 (4.108–13.543)	6.60	＜0.001
2 studies	^123^I-MIBG vs. ^131^I-MIBG	0.0%	0.516	2.032 (1.054–3.918)	2.12	0.034
6 studies	^123^I-MIBG vs. ^18^F-FDG	75.1%	0.001	1.514 (0.491–4.669)	0.72	0.470
1 study	^123^I-MIBG vs ^111^In-pentetreotide	NA	NA	9.486 (3.484–25.826)	4.40	＜0.001
3 studies	^123^I-MIBG vs. CT or MRI	85.1%	0.001	0.115 (0.011–1.170)	1.83	0.068
2 studies	^18^F-FDOPA vs. CT or MRI	0.0%	0.606	10.195 (5.332–19.493)	7.02	＜0.001
2 studies	^18^F-FDG vs. ^131^I-MIBG	0.0%	0.726	1.937 (0.380–9.859)	0.80	0.426
2 studies	CT or MRI vs. ^18^F-FDG	0.0%	0.413	2.674 (1.066–6.705)	2.10	0.036

*Specificity*						
2 studies	^18^F-FDOPA vs. ^123^I-MIBG	3.8%	0.308	3.685 (0.480–28.311)	1.25	0.210
6 studies	^123^I-MIBG vs. ^18^F-FDG	82.0%	0.001	1.007 (0.043–23.643)	0.00	0.996
3 studies	^123^I-MIBG vs. CT or MRI	94.3%	＜0.001	10.378 (0.101–1064.73)	0.99	0.322
2 studies	^18^F-FDOPA vs. CT or MRI	0.0%	0.392	17.906 (5.950–53.884)	5.13	＜0.001
2 studies	^18^F-FDG vs. ^131^I-MIBG	45.9%	0.174	0.269 (0.049–1.496)	1.50	0.134
2 studies	^18^F-FDG vs. CT or MRI	10.0%	0.292	9.435 (5.231–17.019)	7.46	＜0.001

*NPV*						
2 studies	^18^F-FDOPA vs. ^123^I-MIBG	54.6%	0.138	3.255 (0.230–46.060)	0.87	0.383
6 studies	^123^I-MIBG vs. ^18^F-FDG	65.4%	0.013	1.519 (0.538–4.283)	0.79	0.430
3 studies	^123^I-MIBG vs. CT or MRI	90.4%	＜0.001	0.352 (0.014–8.878)	0.63	0.526
2 studies	^18^F-FDOPA vs. CT or MRI	0.0%	0.583	16.819 (7.033–40.218)	6.35	＜0.001
2 studies	^18^F-FDG vs. ^131^I-MIBG	0.0%	0.491	1.038 (0.210–5.126)	0.05	0.964
2 studies	^18^F-FDG vs. CT or MRI	0.0%	0.689	1.472 (0.507–4.277)	0.71	0.477

*PPV*						
2 studies	^18^F-FDOPA vs. ^123^I-MIBG	0.0%	0.816	9.869 (1.722–56.560)	2.57	0.010
6 studies	^123^I-MIBG vs. ^18^F-FDG	80.9%	0.001	0.908 (0.045–18.281)	0.06	0.950
3 studies	^123^I-MIBG vs. CT or MRI	92.8%	＜0.001	3.184 (0.083–122.471)	0.62	0.534
2 studies	^18^F-FDOPA vs. CT or MRI	0.0%	0.388	11.154 (4.216–29.512)	4.86	＜0.001
2 studies	^18^F-FDG vs. ^131^I-MIBG	17.6%	0.271	0.480 (0.100–2.308)	0.92	0.360
2 studies	^18^F-FDG vs. CT or MRI	0.0%	0.354	2.976 (1.774–4.992)	4.13	＜0.001

*DR*						
4 studies	^18^F-FDOPA vs. ^123^I-MIBG	0.0%	0.662	5.616 (3.609–8.739)	7.64	＜0.001
1 study	^123^I-MIBG vs. ^68^Ga-SSAs	NA	NA	0.280 (0.075–1.047)	1.89	0.059
4 studies	^123^I-MIBG vs. ^131^I-MIBG	55.7%	0.079	1.153 (0.464–2.864)	0.31	0.759
8 studies	^123^I-MIBG vs. ^18^F-FDG	91.2%	＜0.001	1.990 (0.842–4.702)	1.57	0.117
1 study	^123^I-MIBG vs ^111^In-pentetreotide	NA	NA	9.486 (3.484–25.826)	4.40	＜0.001
4 studies	CT or MRI vs. ^123^I-MIBG	87.2%	＜0.001	4.654 (1.783–12.150)	3.14	0.002
2 studies	^18^F-FDOPA vs. CT or MRI	80.1%	0.025	1.363 (0.411–4.522)	0.51	0.613
1 study	^68^Ga-SSAs vs. ^131^I-MIBG	NA	NA	1.000 (0.232–4.310)	0.00	1.000
6 studies	^18^F-FDG vs. ^131^I-MIBG	88.7%	＜0.001	0.284 (0.048–1.673)	1.39	0.164
2 studies	CT or MRI vs. ^18^F-FDG	0.0%	0.882	4.599 (2.968–7.126)	6.83	＜0.001
1 study	^131^I-MIBG vs. CT or MRI	NA	NA	3.086 (1.350–7.054)	2.67	0.008

NTs, neuroblastic tumors; NPV, negative predictive value; PPV, positive predictive value; DR, detection rate.

**Table 3:** Subgroup pairwise meta-analysis for DR of NTs.

Included studies	Comparisons	Heterogeneity assessment		Pairwise meta-analysis		
		*I* ^2^	*P*_*h*_	OR (95%CI)	*Z*	*P*

*Primary tumor*						
1 study	^123^I-MIBG vs. ^18^F-FDOPA	NA	NA	0.294 (0.028–3.138)	1.01	0.311
1 study	^123^I-MIBG vs. ^18^F-FDG	NA	NA	2.355 (0.986–5.621)	1.93	0.054
1 study	^123^I-MIBG vs. ^111^In-pentetreotide	NA	NA	9.486 (3.484–25.826)	4.40	＜0.001
1 study	^68^Ga-SSAs vs. ^131^I-MIBG	NA	NA	1.000 (0.232–4.310)	0.00	1.000
2 studies	^18^F-FDG vs. ^131^I-MIBG	0.0%	0.872	1.533 (0.535–4.396)	0.80	0.427

*Bone and bone marrow metastases*						
1 study	^18^F-FDOPA vs. ^123^I-MIBG	NA	NA	5.283 (2.325–12.005)	3.97	＜0.001
1 study	^123^I-MIBG vs. ^131^I-MIBG	NA	NA	0.645 (0.120–3.472)	0.51	0.610
4 studies	^123^I-MIBG vs. ^18^F-FDG	93.5%	＜0.001	2.402 (0.490–11.781)	1.08	0.280
2 studies	CT or MRI vs. ^123^I-MIBG	46.6%	0.171	7.170 (4.503–11.415)	8.30	＜0.001
2 studies	^18^F-FDG vs. ^131^I-MIBG	95.1%	＜0.001	0.339 (0.004–32.575)	0.46	0.642
1 study	CT or MRI vs. ^18^F-FDG	NA	NA	4.525 (2.778–7.353)	6.07	＜0.001

*Primary tumor and metastases*						
2 studies	^18^F-FDOPA vs. ^123^I-MIBG	26.5%	0.244	5.943 (3.480–10.149)	6.53	＜0.001
1 study	^123^I-MIBG vs. ^68^Ga-SSAs	NA	NA	0.280 (0.075–1.047)	1.89	0.059
3 studies	^123^I-MIBG vs. ^131^I-MIBG	66.1%	0.052	1.280 (0.429–3.818)	0.44	0.658
3 studies	^123^I-MIBG vs. ^18^F-FDG	93.3%	＜0.001	1.541 (0.342–6.938)	0.56	0.573
2 studies	CT or MRI vs. ^123^I-MIBG	89.7%	0.002	4.291 (0.813–22.640)	1.72	0.086
2 studies	^18^F-FDOPA vs. CT or MRI	80.1%	0.025	1.363 (0.411–4.522)	0.51	0.613
2 studies	^131^I-MIBG vs. ^18^F-FDG	0.0%	0.797	22.563 (8.020–63.480)	5.90	＜0.001
1 study	CT or MRI vs. ^18^F-FDG	NA	NA	4.926 (1.808–13.333)	3.12	0.02
1 study	^131^I-MIBG vs. CT or MRI	NA	NA	3.086 (1.350–7.054)	2.67	0.008

NTs, neuroblastic tumors; DR, detection rate.

## Data Availability

The data used in this study are available on reasonable request to the corresponding author.

## Abbreviations

NTs: Neuroblastic tumors
PET/CT: Positron-emission tomography/computed tomography
SPECT/CT: Single-photon-emission computed tomography/ computed tomography
CT: Computed tomography
MRI: Magnetic resonance imaging
^18^F-FDG: ^18^F-fluorodeoxyglucose
MIBG: Meta-iodobenzylguanidine
FDOPA: L-3,4-dihydroxy-6-^18^F-fluorophenylalanine
SSAs: Somatostatin analogues
NMA: Network meta-analysis
INRG: International Neuroblastoma Risk Group
EANM: European Association of Nuclear Medicine
PPV: Positive predictive value
NPV: Negative predictive value
DR: Detection rate
TP: True positive
TN: True negative
FP: False positive
FN: False negative
OR: Odds ratio
CI: Confidence interval
PRISMA: Preferred Reporting Items for Systematic Reviews and Meta-Analyses
QUADAS-2: Quality Assessment of Diagnostic Accuracy Studies
P: Primary tumor
BM: Bone and bone marrow metastases
*P* + *M*: Primary tumor and metastases
NETs: Neuroendocrine tumors
PCCs: Pheochromocytoma
^18^F-MFBG: ^18^F-meta-fluorobenzylguanidine
^18^F-FPBG: ^18^F-fluoropropylbenzylguanidine.

## Supplementary Materials

Supplementary Materials The results of publication bias of the subgroup of all data (Figure S1), BM (Figure S2), P (Figure S3), *P* + *M* (Figure S4), and tomographic imaging (Figure S5) are given.
