# Supplementary material for: Diagnostic Value of Seven Different Imaging Modalities for Patients with Neuroblastic Tumors: A Network Meta-Analysis
Source: Contrast Media Mol Imaging. 2021 Sep 1;2021:5333366. doi: 10.1155/2021/5333366 (PMC8429030; doi:10.1155/2021/5333366)
Supplement: Supplementary Materials — The results of publication bias of the subgroup of all data (Figure S1), BM (Figure S2), P (Figure S3), P + M (Figure S4), and tomographic imaging (Figure S5) are given. [file 5333366.f1.docx]

Supplementary Materials for

Diagnostic Value of Seven Different Imaging modalities for Patients with Neuroblastic Tumors: A Network Meta-Analysis

Yu Wang^1^ · Yanfeng Xu^1^ · Ying Kan^1^ · Wei Wang^1^ · Jigang Yang^1^

^1^ Department of Nuclear Medicine, Beijing Friendship Hospital, Capital Medical University, Beijing 100050, China

Corresponding author: Jigang Yang^1^

E-mail: yangjigang@ccmu.edu.cn

Tel: 18811695983


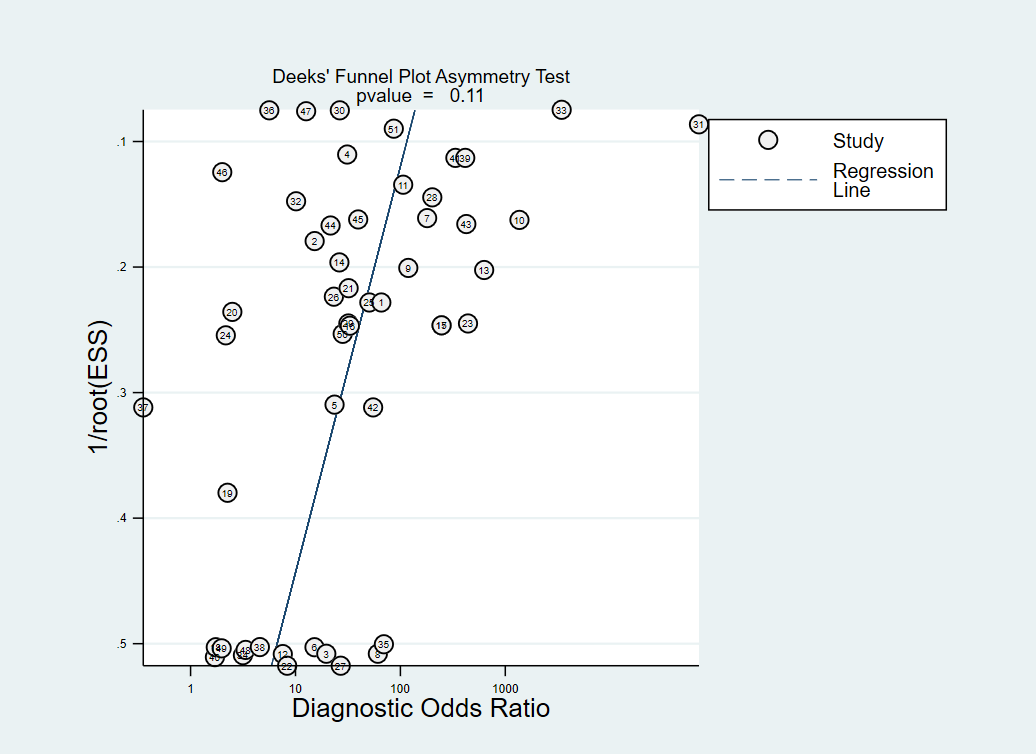


Figure S1: the result of publication bias of all data.


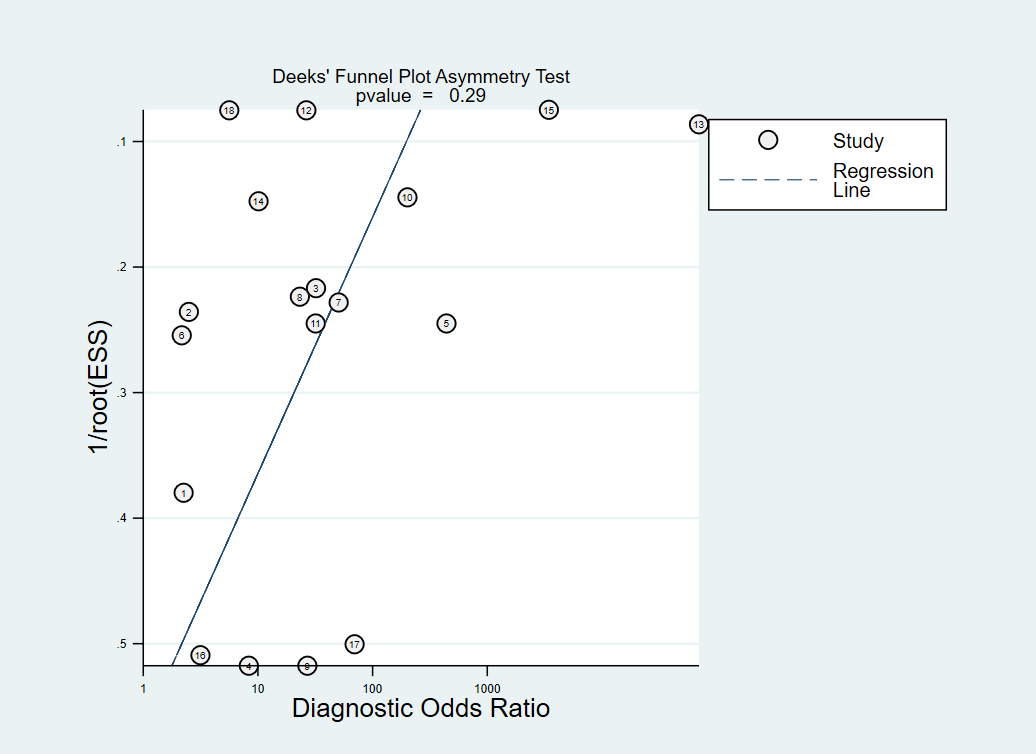


Figure S2: the result of publication bias of the subgroup of BM


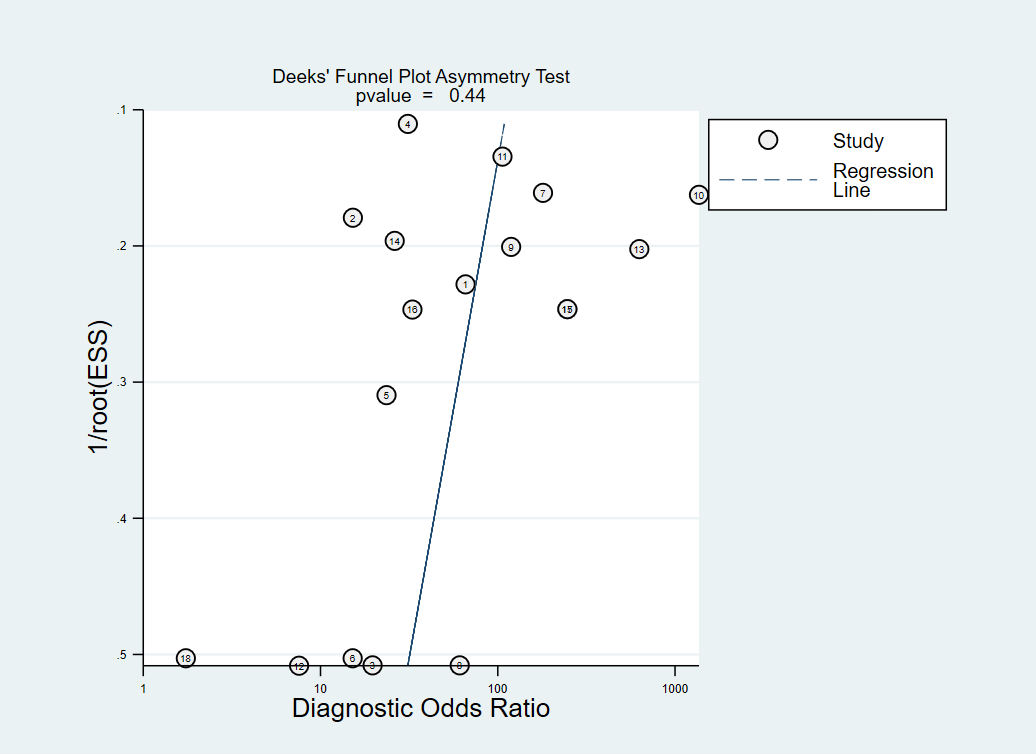


Figure S3: the result of publication bias of the subgroup of P.


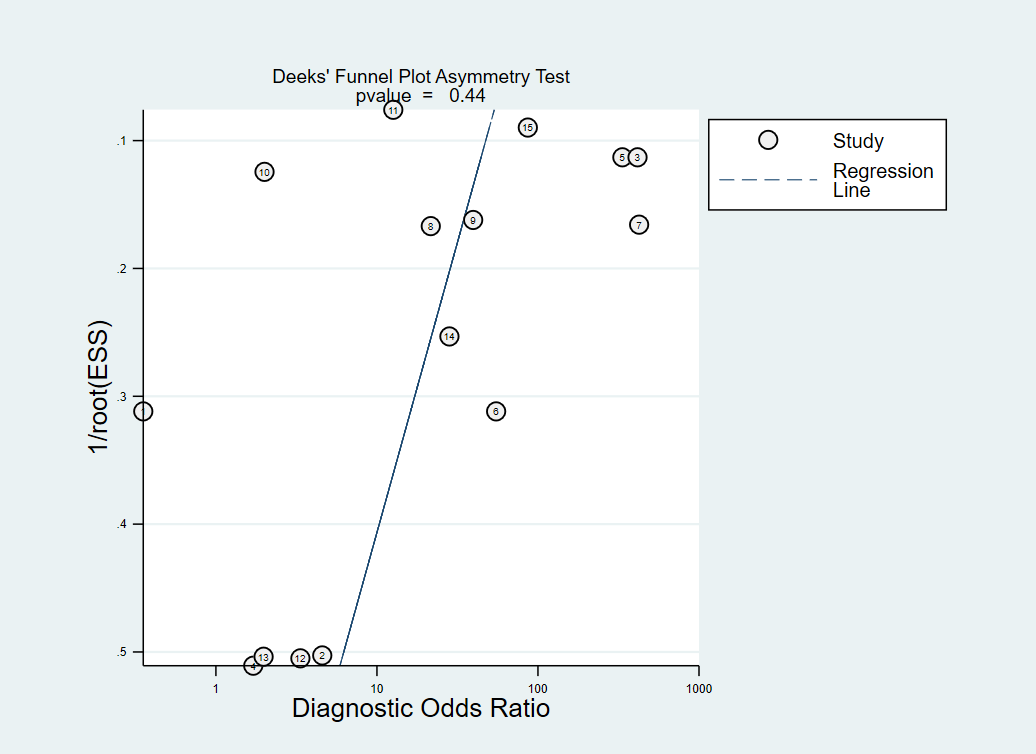


Figure S4: the result of publication bias of the subgroup of P+M.


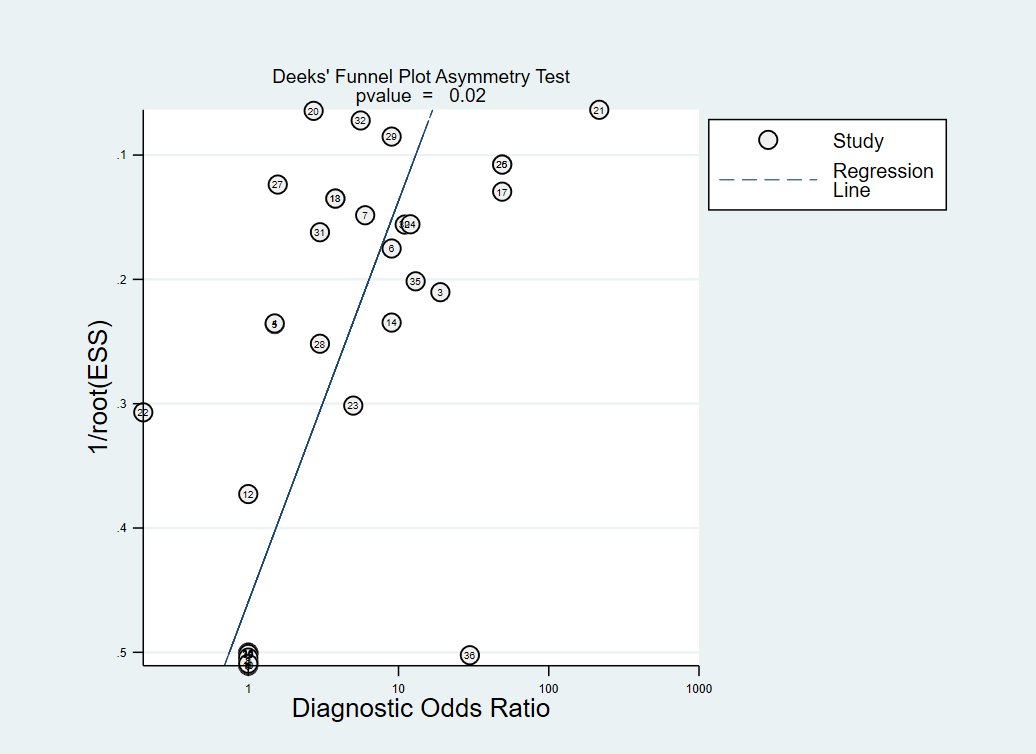


Figure S5: the result of publication bias of the subgroup of Tomographic imaging.
